# Sharp drop in under-five mortality and associated factors in Burkina Faso from 2010 to 2021: is it a composition or performance effect?

**DOI:** 10.3389/fpubh.2025.1549628

**Published:** 2025-04-28

**Authors:** Hervé Bassinga, Sibiri Clément Ouedraogo, Aristide Romaric Bado, Herman Bazié, Kadidia Kouadima Diallo, Yacouba Savadogo

**Affiliations:** ^1^Institut Supérieur des Sciences de la Population (ISSP), Université Joseph Ki-Zerbo, Ouagadougou, Burkina Faso; ^2^Institut National de la Statistique et de la Démographie, Ouagadougou, Burkina Faso; ^3^Institut de Recherche en Sciences de la Santé (IRSS/CNRST), Ouagadougou, Burkina Faso; ^4^Institut National de Santé Publique (INSP), Ouagadougou, Burkina Faso

**Keywords:** under-five mortality, drop, performance effect, composition effect, Burkina Faso

## Abstract

**Background:**

Long maintained above 100 ‰ since the 1960s, under-five mortality rates in Burkina Faso have experienced a significant decline, although the specific sources of change remain difficult to identify. Notably, under-five mortality increased from 187 ‰ in 1993 to 219 ‰ in 1998. Subsequently, a steady decrease was observed, with rates falling to 184 ‰ in 2003, 129 ‰ in 2010, and ultimately reaching 48 ‰ in 2021.

**Objectives:**

The aim of this study was to analyze the sources of change and the factors that have contributed to the decline in under-five mortality rates in Burkina Faso from 2010 to 2021.

**Method:**

Utilizing data from Demographic and Health Surveys (DHS), this analysis encompassed the period from 2010 to 2021 and used an Oaxaca-Blinder multivariate decomposition method. This approach facilitated the differentiation of the components of change that can be attributed to the demographic composition (structure) of the population versus those attributable to behavioral changes and policy effectiveness. The analysis samples consisted of 15,045 and 12,242 children in 2010 and 2021, respectively.

**Results:**

The results showed that the observed decline in under-five mortality was largely attributable to changes in unobserved variables rather than to changes in population structure. Specifically, changes in population structure accounted for only 23% of the decline in under-five mortality between 2010 and 2021, while the effect of overall variation in coefficients related to the efficacy of control measures and behavioral changes accounted for 77% of the decline. Among the factors associated with performance and behavioral changes, the involvement of unemployed women (13.1%) has significantly contributed to the reduction of child mortality rates.

**Conclusion:**

Urbanization has led to a decline in under-five mortality rates, likely attributable to improved access to health services. Therefore, regions such as the Sahel and the Southwest, which have high mortality rates, should be prioritized for targeted intervention. It is imperative to enhance healthcare provision in rural areas to mitigate disparities. To maximize the efficacy of these interventions, a holistic approach that encompasses advancements in education, economic development, and sanitation is essential. In conjunction with development efforts, behavior change communication constitutes a critical lever in the fight against child mortality.

## Highlights

- The decline in under-five mortality is largely attributable to changes in unrecorded variables rather than to changes in population structure.
- Changes in population structure collectively explain 23% of the decline in under-five mortality between 2010 and 2021.
- The effect of the overall variation in coefficients attributable to the performance of actions to combat mortality and to changes in behavior explains 77% of the decline in under-five mortality between 2010 and 2021.
- In terms of performance and behavioral changes, it is important to note the action of unemployed women (13.1%), who have succeeded in significantly reducing the level of under-five mortality.

## 1 Introduction

Data concerning under-five mortality is crucial for the demographic evaluation of a population and serves as a significant indicator of a nation’s socio-economic development and quality of life. Such information facilitates the identification of social strata in which children are most vulnerable to mortality, thereby assisting in the formulation and implementation of strategies aimed at reducing child mortality rates. In Burkina Faso, while current neonatal and under-five mortality rates remain among the highest in the world (1) and national as well as international targets in this domain are not being achieved, it is noteworthy that these rates have experienced substantial declines over recent decades. Specifically, neonatal mortality has consistently decreased since 1993, from 43 ‰ to 18 ‰ in 2021. In terms of infant mortality, the decline has been somewhat less uniform; the rate increased from 94 ‰ to 105 ‰ between 1993 and 1998–99, followed by a steady decrease to 30 ‰ in 2021. Overall, under-five mortality, which rose from 187 ‰ to 219 ‰ between 1993 and 1998, subsequently entered a period of continuous decline, decreasing to 184 ‰ in 2003, then to 129 ‰ in 2010, and ultimately reaching 48 ‰ in 2021 (2).

The consistent reduction in all components of under-five mortality since 2000 can be attributed, in part, to the numerous actions and initiatives implemented by the government of Burkina Faso in collaboration with its development partners. The adoption of the Millennium Development Goals (MDGs) in 2000, followed by the Sustainable Development Goals (SDGs) in 2015, prioritized the fight against child mortality, specifically through MDG 4 and SDG 3.

In addition to these international commitments, Burkina Faso and other African countries have engaged in various initiatives under the New Partnership for Africa’s Development (NEPAD) and the African Union’s Declaration on Child Survival, aimed at enhancing the continuous monitoring of the concerning status of children’s health (3, 4). Furthermore, in conjunction with these international initiatives to which the country has subscribed, there are the Strategy for Accelerated Growth and Sustainable Development (SCADD), the National Economic and Social Development Plan (PNDES), and the National Health Development Plan (PNDS), which are implemented through national development programs.

As a result, numerous initiatives have been implemented to alleviate poverty, combat malaria, address malnutrition, and tackle both communicable and non-communicable diseases, while also enhancing obstetric, pre- and post-natal care, as well as access to such care services. A pivotal measure introduced by the government of Burkina Faso in 2016 was the provision of free healthcare for children under the age of five and pregnant women aimed at eliminating financial barriers to healthcare access. This policy, which took effect in April 2016, has likely contributed to the noteworthy reduction in child mortality rates observed in recent years; however, the specific factors that have influenced this decline remain unclear. For example, a study by Aye and co-authors on the effect of free healthcare on facility deliveries and curative care for children under five, antenatal care, and curative care for children over five, found that the monthly utilization rate among children under five compared with those over five (controls) immediately increased by 111.19 visits per 1,000 children due to the gratuity. This immediate effect then declined with a monthly change of 0.93 per 1,000 children (5). Consequently, it is imperative to investigate the determinants of the decrease in under-five mortality in Burkina Faso over the past two decades, with the intention of equipping policymakers with actionable insights to sustain and enhance the ongoing reduction in mortality within this population group.

The primary objective of this study is to enhance the understanding of the factors associated with the decline in under-five mortality in Burkina Faso, thereby providing valuable insights for policymakers to facilitate informed decision-making aimed at further accelerating the reduction of child mortality rates. Specifically, this study seeks to identify the subpopulations that have experienced significant reductions in child mortality and to ascertain the principal sources contributing to the decline in under-five mortality between 2010 and 2021.

Several approaches have been employed by various authors to comprehend and elucidate the phenomena of infant and child mortality. These approaches highlight five categories of factors that are likely to explain under-five mortality: socio-economic factors, biodemographic factors, socio-cultural factors, environmental factors, and maternal behavior-related factors (6–21). However, analysis of the factors and sources associated with changes in child mortality is rare (15, 17, 21–24).

The meta-analysis carried out by Gakidou et al. (25), compiling data from 915 censuses and national surveys conducted between 1970 and 2009, shows a negative correlation between the average level of education of women of childbearing age and the rates of infant and child mortality in 175 countries. Between 1970 and 2009, 51% of the reduction in under-five mortality in the countries studied was attributed to improvements in women’s education. The effect of educational development on children’s health has been significant; in fact, it is estimated that 4.2 million deaths have been averted due to advancements in women’s education (25). More recently, Ayipe and Tanko (17) concluded, using panel data from 2000 to 2019, that domestic health expenditure has a significant negative relationship with the under-five mortality rate in low-income countries in sub-Saharan Africa. For every additional percentage increase in domestic health expenditure, it is likely to result in a decrease of about 5.3 units in the under-five mortality rate. Additionally, high fertility, female population, and rural populations practicing open defecation were positively associated with under-five mortality.

Van Malderen et al. (21) analyzed the main contributors to under-five mortality based on reproductive history from Demographic and Health Surveys (DHS) conducted in 32 sub-Saharan African countries between 2010 and 2016. The relative contribution of child sex, place of residence, maternal education level, and household wealth level to the variability of under-five deaths was assessed using a regression decomposition of a Gini-type index. The results revealed that the main contributors to the Gini index (with a relative contribution greater than 25%) were different across countries: maternal education level in 13 countries, child sex in 12 countries, household wealth in 11 countries, and place of residence in 8 countries (in some countries, more than one main contributor was identified).

Using the multivariate decomposition method, Bassinga and Soura demonstrate that the decline in child mortality observed in Burkina Faso between the 1996 and 2006 censuses can primarily be attributed to alterations in the demographic and health behaviors of the population, along with the effectiveness of health policy interventions. In addition to the predominant influence of performance and behavioral effects, an analysis of the sources of change in child mortality across various provinces offers further affirmation that advancements in maternal education and improvements in household living conditions consequently contributed to a reduction in child mortality (26).

The decline in infant mortality in Burkina Faso between 2003 and 2010, as evidenced by data from the DHS, can be attributed predominantly to substantial improvements in rural areas. The findings derived from the decomposition method underscore the critical impact of health system performance, which contributed to the reduction in mortality rates. Conversely, the effect of social composition has served to exacerbate mortality rates in rural regions (27).

In Ghana, a multivariate decomposition analysis showed that the observed decline in infant and under-five mortality rates could be attributed to an increased proportion of children sleeping under mosquito nets. In addition, a yearly increment in the percentage of women participating in the labor market correlates with a reduction of 10 and 18 infant and under-five deaths per 1,000 live births, respectively (23).

In conclusion, the literature review shows that infant and child mortality cannot be examined in isolation, particularly in the context of developing countries, due to the myriad of factors influencing this phenomenon. The determinants of infant and child mortality encompass socio-economic, cultural, environmental, socio-demographic, and behavioral factors. Hence, this study adopts a comprehensive approach that considers the various contributing factors.

## 2 Materials and methods

### 2.1 Study area

Located in the center of West Africa, Burkina Faso is a landlocked country that shares its borders with six countries: Mali to the north and west, Niger to the east, Côte d’Ivoire, Togo, Ghana and Benin to the south. The country has a tropical, dry, and harsh Sudano-Sahelian climate, characterized by considerably variable rainfall ranging from 350 mm in the northern part of the country to more than 1,000 mm in its southwestern part. This climatic variability, which is more or less erratic from one geographical area to another, impacts both the availability of water resources for agricultural production and children’s health. Demographically, with a high fertility rate, the country has always been characterized by the youth of its population. The under-15 age group has continuously represented more than 45% of the total population (28). There is, therefore, still a burden to address and meet basic social needs. On the socioeconomic front, enormous challenges remain. In 2019, only 29.7% of the Burkinabe population was literate (28). As for the incidence of monetary poverty, it has not declined significantly for decades, rising from 44.5% in 1994 to 47% in 2009 and then to 43.2% in 2021 (29).

As for under-five mortality, it has been steadily declining. It fell from 187 deaths per 1,000 births in 1993 to 129 deaths per 1,000 births in 2010, and then to 48 deaths per 1,000 in 2021 (2). Although this mortality rate is continuously declining, it remains high and far from the target of 25‰ set in the SDGs by 2030. Its infant and juvenile components also experienced a decrease during the same period, falling from 94‰ to 30‰ and from 103‰ to 18‰, respectively. Malaria and respiratory infections are, in fact, the main causes among death of children under five, both in routine data from the Ministry of Health and in population observatories (20, 30, 31). These two types of diseases are responsible for more than half of child deaths in the Ouagadougou Population Observatory (30).

### 2.2 Data sources

The data used in this study are derived from the DHS carried out in Burkina Faso in 2010 and 2021. The two operations were conducted, respectively, from May 20, 2010, to January 15, 2011, and from July 30 to November 30, 2021. DHS data offer comprehensive information on various topics, including mortality rates and the socio-demographic characteristics of both children and their mothers. These surveys samples are representative at the national level, as well as at the level of regions and places of residence (urban vs. rural).

DHS are household surveys based on a random, stratified, two-stage sampling design. The primary sampling unit is the cluster. Each region was subdivided into urban and rural components to create the sampling strata, with independent sampling conducted in each stratum. A total of 26 strata were established. In the first stage of sampling in 2010, 574 clusters were selected with probability proportional to size. In 2021, this number was 514 clusters. In the second stage of the sampling, 26 households were systematically chosen with equal probability from each of the clusters identified in the first stage. The survey involved a representative sample of 17,087 women aged 15–49 in 2010 and 17,659 women in 2021, respectively. Data were collected through face-to-face interviews utilizing structured questionnaires (paper-based in 2010, CAPI in 2021).

### 2.3 Ethical considerations

This study analyzed secondary data without including any information that could identify participants. Ethically, the protocols for the two DHSs received favorable approval and various administrative authorizations. Data collection, processing, and analysis were conducted in strict adherence to ethical principles, particularly regarding the investigators’ health agreements, confidentiality, and anonymity.

### 2.4 Study population

The study population included live births during the 5 years preceding each survey. There were 15,045 live births in 2010 and 12,242 in 2021. Data on these births were obtained from the pregnancy histories of women of childbearing age (15–49).

### 2.5 Study variables

The dependent variable is the child’s death status. It has two modalities: 1 if the child has died and 0 if the child is still alive at the time of the survey. The following explanatory variables were used: region, place of residence, children sleeping under mosquito nets, mother’s level of education, mother’s religion, mother’s ethnicity, mother’s occupation, child’s sex, mother’s age at delivery, birth order, and standard of living. This last variable was obtained by principal component analysis from the goods and equipment owned by households. We classified households into five categories according to quartiles: very poor, poor, average, rich, and very rich.

### 2.6 Statistical analysis

The analytical method used in this study is based on Oaxaca-Blinder’s multivariate decomposition, as summarized by Powers et al. (32). We use this method to identify sources of decline in under-five mortality. This method aims to quantify the contributions of the different analysis variables to differences in mean predictions between two groups based on multivariate regression models. Specifically, the technique leverages the coefficients derived from a regression model to estimate the proportion of statistical differences (either mean or proportion) between the two groups that can be attributed to disparities in population characteristics, as well as the proportion attributable to behavioral or performance differences (32). Additionally, this technique can be applied in a longitudinal framework to decompose changes in a phenomenon into components associated with performance effect and a component linked to changes in the composition of the population studied (composition effect). For the aggregate level analysis, we used a linear prediction function, given the quantitative nature of the variables under consideration.

Let Y be the N x 1 vector of the dependent variable, X the N x K matrix of independent variables, and *β* a K x 1 vector of regression coefficients. The difference in the means of Y between groups A and B (with A and B representing, in our case, the years 2010 and 2021, respectively) can be written as a function of the independent variables and the regression coefficients obtained separately in groups A and B (Equation 1).


(1) $${\bar{Y}}_{A}-{\bar{Y}}_{B}=F\left(X_{A}\beta_{A}\right)-F\left(X_{B}\beta_{B}\right)$$

An arrangement of Equation 1 allows the difference to be broken down as follows:


(2) $${\bar{Y}}_{A}-{\bar{Y}}_{B}=\underset{E}{\underset{︸}{\bar{F\left(X_{A}\beta_{A}\right)}-\bar{F\left(X_{B}\beta_{A}\right)}}}+\underset{C}{\underset{︸}{\bar{F\left(X_{B}\beta_{A}\right)}-\bar{F\left(X_{B}\beta_{B}\right)}}}$$

This arrangement (Equation 2) is called twofold decomposition in that it splits the average difference into two components (32, 33). The E component refers to the part of the differential linked to disparities in characteristics, generally referred to as the explained effect or the characteristics effect. The C component refers to the part of the differential attributable to differences in the coefficients, usually called the unexplained component or coefficient effect. In this equation, group A is considered the control group. Thus, E reflects a counterfactual comparison of the difference in results from the point of view of group A, i.e., the difference expected if group B were given the coefficients of group A. Thus, the coefficients remain identical for both groups; only the characteristics vary. Component C reflects a counterfactual comparison of the results from the point of view of group B, i.e., the difference expected if the characteristics of group B were applied to group A. In the latter case, the two groups have the same characteristics and different coefficients.

By setting the coefficients of the composition component (E) to Group A levels, we estimate the contribution to the differential that would have occurred if the effects associated with the characteristics had been set to Group A values. By setting the characteristics to Group B levels in component C, we assess the contribution to the differential that is due to the difference in effects. This difference in effects is also known as the unexplained difference since the differences in observable characteristics do not allow us to account for it (34). It is often attributable to a difference in performance between the two groups, understood in the sense of changes in demographic and health behaviors and in the efficiency of the health sector. This difference in coefficients, like the composition effect, may, of course, be influenced by characteristics not taken into account by the model.^1^

The above equations do not allow us to understand the unique contribution of each predictor to each component of the difference. To do this, a detailed description of the method is required. The E and C components can be partitioned into Ek and Ck portions (*k* = 1,…, K), which represent the contribution of each variable to E and C, respectively (Equations 3 and 4). These portions are obtained using the following formulae:


(3) $$E_{k}=W_{\Delta x_{k}}E$$

(4) $$C_{k}=W_{\Delta \beta_{k}}C$$

Thus, in a linear model, the weights of the E component, i.e., the $W_{\Delta x_{k}}$ are obtained by the following formula in which ${\bar{X}}_{Ak}$ and ${\bar{X}}_{Bk}$ are the means of X_k_ in groups A and B, respectively, and β_Ak_ is the coefficient of the variable Xk in group A (Equation 5).


(5) $$W_{\Delta_{x_{k}}}=\frac{\beta_{A_{k}}\left({\bar{X}}_{A_{k}}-{\bar{X}}_{B_{k}}\right)}{\overset{K}{\underset{k=1}{\Sigma }}\beta_{A_{k}}\left({\bar{X}}_{A_{k}}-{\bar{X}}_{B_{k}}\right)}$$

As for the weighting coefficients of component C, that is to say the $W_{\Delta \beta_{k}}$ , they are obtained by the formula below, in which ${\bar{X}}_{Ak}$ denotes the average of X_k_ in group A. β_Ak_ and β_Bk_ are, respectively, the coefficients of the variable X_k_ in group A and in group B (Equation 6).


(6) $$W_{\Delta_{\beta_{k}}}=\frac{{\bar{X}}_{A_{k}}\left(\beta_{A_{k}}-\beta_{B_{k}}\right)}{\overset{K}{\underset{k=1}{\Sigma }}{\bar{X}}_{A_{k}}\left(\beta_{A_{k}}-\beta_{B_{k}}\right)}$$

For each component, the weighting coefficients are such that their sum over all the variables is equal to 1.

The composition weights $W_{\Delta_{x_{k}}}$ are a function of the magnitude of each group’s specificity in the characteristics, with the latter being weighted by the mean effects of the reference group. Similarly, the coefficient weights $W_{\Delta_{\beta k}}$ are a function of the magnitude of group disparity in the effects (coefficients), with the latter being weighted by the mean of the characteristics of the control group.

The raw difference can now be expressed as a weighted sum of the unique contributions (Equation 7).


(7) $${\bar{Y}}_{A}-{\bar{Y}}_{B}=E+C=\underset{k}{\Sigma }W_{\Delta_{x_{k}}}E+\underset{k}{\Sigma }W_{\Delta_{\beta_{k}}}C=\Sigma_{k=1}^{k}E_{k}+\Sigma_{k=1}^{k}C_{k}$$

In practice, Jann (33) proposed the Oaxaca command that allows the application of such a decomposition in the Stata software. A few years later, Powers and his colleagues (35) made improvements through another command called *mvdcmp* (multivariate decomposition). This command is applicable to both linear and nonlinear models. In the case of the linear model, in addition to providing the same coefficients as the Oaxaca command, *mwdcmp* offers the percentage contributions of the variables and the blocks of variables. We have exploited this more recent command.

For the case of the linear prediction function, the significance of the contributions is tested in the classical way, which exploys the Student’s t statistic, obtained by relating each coefficient to its standard deviation. These standard deviations are estimated using a method called Delta. For more details, see Rao (35).

## 3 Results

### 3.1 Study samples description

Table 1 shows the changes in the structure of the population between 2010 and 2021. There is a variation in the composition according to several characteristics, such as place of residence and the education of woman. The percentage of the population living in urban areas increased from 16.8% in 2010 to 24.6% in 2021. The proportion of women with formal education decreased by 10 percentage points.

**Table 1:** Distribution of children under 5 years of age according to certain characteristics in 2010 and 2021.

Variables	2010		2021		Total	
	Percentage	Effective	Percentage	Effective	Percentage	Effective
Type of place of residence						
*Urban*	16.8	2576	24.6	3002	20.2	5579
*Rural*	83.2	12799	75.4	9208	79.8	22007
Region						
*Boucle du Mouhoun*	11.9	1827	9.8	1199	11	3027
*Cascades*	3.7	570	3	369	3.4	939
*Centre*	8.4	1290	15.3	1873	11.5	3163
*Centre-Est*	7.7	1186	10.9	1333	9.1	2519
*Centre-Nord*	7.9	1210	9.9	1211	8.8	2421
*Centre-Ouest*	8	1228	10.2	1243	9	2471
*Centre-Sud*	4.4	673	4	487	4.2	1161
*Est*	11.3	1742	8.1	985	9.9	2726
*Hauts Basins*	10.7	1652	9.7	1180	10.3	2832
*Nord*	7.5	1160	6.2	753	6.9	1913
*Plateau Central*	4.5	685	6.1	743	5.2	1428
*Sahel*	9.5	1467	3.1	375	6.7	1841
*Sud-Ouest*	4.5	684	3.8	460	4.1	1144
Wealth index						
*Poorest*	20.8	3194	21.2	2593	21	5787
*Poorer*	21.8	3359	20.8	2537	21.4	5895
*Middle*	21.7	3338	20.9	2548	21.3	5886
*Richer*	20.7	3183	19.8	2423	20.3	5606
*Richest*	15	2301	17.3	2110	16	4411
Children under 5 slept under mosquito bed net last night						
*No*	39.2	5886	21.2	2529	31.2	8415
*All children*	47.2	7093	59.7	7113	52.7	14206
*Some children*	13.6	2044	19.1	2271	16	4315
Highest educational level						
*No education*	84.3	12963	70	8545	78	21508
*Primary*	10.6	1630	13.4	1641	11.9	3271
*Secondary*	5	776	16.6	2024	10.1	2799
Religion						
*Muslim*	63.8	9788	66.2	8078	64.9	17866
*Catholic*	26.6	4086	29.7	3629	28	7715
*Protestant*	9.5	1463	4.1	503	7.1	1966
Ethnicity						
*Mossi*	50	7677	54.5	6658	52	14336
*Bobo/Dioula*	5.4	830	3.6	437	4.6	1267
*Fulfulde/peulh/Touareg/Bella*	12.2	1874	7.6	927	10.2	2800
*Gourmantche*	9.2	1418	6.5	797	8	2215
*Gourounsi*	4.3	653	6	727	5	1380
*Lobi/Dagara*	4.6	700	3	364	3.9	1064
*Senoufo*	4.5	698	2.2	270	3.5	967
*Bissa*	3.6	560	5.4	661	4.4	1221
*Others*	6.1	941	11.2	1369	8.4	2310
Respondent’s occupation (grouped)						
*Not working*	18.4	2821	35.6	4325	26	7146
*Professional/technical/managerial*	81.6	12474	64.4	7837	74	20311
Mother age at birth						
<20 years	14.8	2269	14.6	1780	14.7	4049
20–29 years	52.2	8028	49.3	6016	50.9	14045
30–39 years	28.3	4356	31.1	3800	29.6	8156
40–49 years	4.7	722	5	615	4.8	1336
Sex of child						
*Male*	50.7	7800	50.9	6218	50.8	14018
*Female*	49.3	7575	49.1	5993	49.2	13568
Birth order						
*1*	18.5	2847	21.5	2627	19.8	5474
*2*	32.9	5056	36.1	4410	34.3	9467
*3*	33.4	5142	33.6	4102	33.5	9244
*4*	15.2	2330	8.8	1071	12.3	3401
Total	100	15375	100	12210	100	27585

### 3.2 Analysis of the evolution of the under-five mortality rate between 2010 and 2021

The significant decline in under-five mortality between 2010 and 2021 is observed in almost all socioeconomic, demographic, and geographic categories. Indeed, from 2010 to 2021, the under-five mortality rate fell from 137.5‰ to 52.5‰ in rural areas and from 81.7‰ to 33.3‰ in urban areas. With the exception of the Cascades and Centre-Est regions, there was a significant drop in under-five mortality in all regions from 2010 to 2021. The most significant declines in the mortality rate from 2010 to 2021 were recorded in the Sahel and Est regions, with 114.3 points and 111.4 points, respectively. The smallest decrease was recorded in the Centre-East and Centre-North regions, with 20.8 points and 47.8 points, respectively. This decrease is remarkable among very poor and poor households, where the under-five mortality rate decreased by 102.6 points and 87.9 points, respectively. The same is true for the decrease according to the mother’s level of education. The higher the mother’s level of education, the lower the decrease in the mortality rate. It is 85.6 points among mothers who have no level of education, 41.5 points among mothers who have a primary level of education, and 25.5 points for mothers who have a secondary level and above. This could be explained by the fact that under-five mortality was higher among mothers who have no level of education and those who have a primary level of education.

### 3.3 Analysis of the results of the multivariate decomposition

As for changes related to the structure of the population (composition), they contribute positively and significantly to the decline in mortality. Indeed, the changes in the structure of the population observed according to the different explanatory variables collectively explain 23% of the decline in under-five mortality between 2010 and 2021. The effect of the overall variation in coefficients on the decline in mortality is also significant. This effect, attributable to the performance of control actions and changes in behavior, explains 77% of the decline. The decline in mortality among children under five is thus largely attributable to changes in behavior rather than to changes in the structure of the population (see Table 2).

**Table 2:** Evolution of the under-five mortality rate between 2010 and 2021.

Variables	Modality	2010	2021	Diff
Total	Together	128.4 [121.2–136.1]	47.9 [43.6–52.6]	80.5***
Place of residence	Urban	81.7 [70.5–94.5]	33.3 [27.0–40.9]	48.4***
	Rural	137.5 [129.2–146.3]	52.5 [47.2–58.3]	85.1***
Region of residence	Boucle du Mouhoun	119.2 [93.2–151.2]	47.1 [36.1–61.3]	72.0***
	Cascades	127.7 [103.8–156.0]	76.4 [44.7–127.6]	51.3 ns
	Centre	79.0 [60.9–101.9]	27.1 [19.9–37.0]	51.9***
	Centre–Est	74.8 [61.2–91.1]	54.0 [38.9–74.5]	20.8 ns
	Centre–Nord	101.2 [78.2–130.0]	53.4 [42.1–67.6]	47.8**
	Centre–Ouest	119.7 [97.6–146.0]	37.2 [25.3–54.3]	82.5***
	Centre–Sud	112.6 [94.0–134.4]	47.3 [32.8–67.7]	65.4***
	Est	159.4 [134.0–188.6]	48.0 [36.8–62.3]	111.4***
	Hauts–Bassins	115.7 [96.5–138.2]	50.7 [38.2–67.0]	65.0***
	Nord	132.4 [109.7–158.9]	38.6 [27.3–54.3]	93.8***
	Plateau Central	123.6 [100.5–151.0]	37.9 [27.0–53.0]	85.6***
	Sahel	207.2 [178.9–238.7]	92.9 [48.0–172.1]	114.4**
	Sud–Ouest	192.8 [161.9–227.9]	94.3 [75.1–117.8]	98.5***
Standard of living	Very poor	149.0 [133.4–166.1]	61.1 [50.7–73.5]	87.8***
	Poor	157.0 [140.8–174.6]	54.4 [45.2–65.5]	102.5***
	Average	127.7 [115.3–141.2]	51.0 [42.3–61.3]	76.7***
	Rich	112.7 [99.7–127.2]	37.8 [30.3–47.2]	74.9***
	Very rich	78.3 [65.3–93.5]	30.4 [23.2–39.7]	47.9***
Children in the household who slept under mosquito nets	None	107.4 [98.1–117.5]	40.1 [32.2–50.0]	67.3***
	All	122.6 [112.7–133.2]	41.0 [36.0–46.7]	81.6***
	Some	104.8 [88.9–123.2]	41.7 [32.8–52.8]	63.1***
education level	None	136.8 [128.7–145.3]	51.2 [46.0–56.9]	85.6***
	Primary	89.8 [75.1–107.0]	48.3 [37.7–61.7]	41.4***
	Secondary and above	55.8 [39.6–78.1]	30.3 [23.0–39.8]	25.5*
Mother’s occupation	Inactive	153.0 [132.9–175.6]	46.0 [38.6–54.6]	107.0***
	Active	123.1 [115.6–130.9]	49.1 [44.1–54.6]	74.0***
Mother’s religion	Muslim	130.4 [121.3–140.0]	48.1 [42.4–54.5]	82.3***
	Christian	107.9 [96.7–120.3]	43.1 [36.4–51.0]	64.8***
	Others	171.9 [148.5–198.0]	77.6 [60.8–98.5]	94.3***
Mother’s ethnicity	Mossi	113.8 [104.7–123.6]	42.1 [37.1–47.7]	71.8***
	Bobo/Dioula	107.6 [83.1–138.1]	32.7 [21.1–50.3]	74.9***
	Fulfulde/Peulh/Touareg/Bella	165.1 [141.3–192.0]	56.5 [35.0–90.0]	108.5***
	Gourmantché	170.1 [145.0–198.6]	53.4 [40.9–69.4]	116.8***
	Gourounsi	115.3 [81.1–161.4]	41.1 [27.9–60.3]	74.2***
	Lobi/Dagara	184.9 [151.9–223.2]	90.8 [70.6–116.0]	94.1***
	Senoufo	140.0 [109.6–177.2]	76.2 [41.1–136.7]	63.9 ns
	Bissa	74.3 [55.6–98.7]	51.6 [31.8–82.7]	22.7 ns
	Others	118.9 [96.8–145.3]	57.1 [45.0–72.2]	61.8***
Mother’s age at birth	<20 years	151.7 [134.3–170.8]	50.8 [40.7–63.3]	100.8***
	20–29 years	120.9 [112.2–130.2]	42.0 [36.7–48.0]	78.9***
	30–39 years	126.0 [114.4–138.5]	48.2 [40.9–56.7]	77.8***
	40–49 years	152.0 [120.3–190.2]	98.5 [73.7–130.3]	53.5*
Sex of the child	Male	132.8 [123.2–143.1]	53.2 [47.1–60.1]	79.6***
	Female	123.9 [115.2–133.3]	42.4 [36.6–49.0]	81.6***
Birth order	1	124.6 [110.5–140.2]	39.7 [32.4–48.6]	84.9***
	2–3	117.6 [106.7–129.5]	40.0 [34.0–47.0]	77.6***
	4–6	125.1 [115.0–136.0]	46.8 [40.4–54.2]	78.2***
	7+	164.8 [147.1–184.1]	104.0 [83.5–128.8]	60.8***

Significance level: *** 1%; ** 5%; * 10%; ns, not significant.

Table 3 highlights, for each variable, its coefficient and its percentage contribution, attributable on the one hand to changes in characteristics (composition) and on the other hand to changes in coefficients (performance effect, behavior effect).

**Table 3:** Overall result of the multivariate decomposition of the decline in infant and child mortality.

Death	Coefficient (Coef)	*p* > z	Percentage (Pct)
Part due to characteristics (E)	0.01040	0.000	23.39
Part due to coefficients (C)	0.03407	0.000	76.61
Change	0.04448	0.000	

#### 3.3.1 Composition effect

In terms of composition effects (changes in the structure of the population), all variables except the standard of living and the mother’s employment status contributed significantly to the decline in under-five mortality. As shown in Table A1 in the appendix, the distribution of children according to standard of living quintiles changed very little between the 2 years. As for the distribution according to employment status, it changed to the disadvantage of working mothers (81.6% in 2010 compared to 64.4% in 2021) but had no impact on infant and child mortality.

Furthermore, changes in the composition of the population according to place of residence accounted for 4.5% of the decline in under-five mortality between 2010 and 2021. In other words, urbanization is one of the drivers of this decline. The distribution of children by region has changed between 2010 and 2021. Some regions have seen their shares decrease while others have increased. The increase in the proportion of children residing in the Centre-East (a region with relatively low mortality) is associated with 2.2% of the decrease in under-five mortality. Additionally, the decrease in the proportion of children residing in certain regions at high risk of under-five mortality, such as the Sahel and the South-West, contributed to the decrease by around 3.9 and 0.6%, respectively.

In addition, the increase in the proportion of households where some children sleep under mosquito nets is responsible for 2.2% of the decrease. Similarly, 5.3% of the overall decrease in under-5 mortality is attributable to the decrease in the proportion of uneducated mothers.

According to ethnicity, the decrease in the proportion of Gourmantché children is associated with 1.8% of the decrease in under-five mortality. Changes in the age structure of mothers contributed to the decrease in mortality, particularly the under-20 and 30–39 age groups, which contributed 0.2 and 0.9%, respectively. Also, the slight decrease in the proportion of girls from 49.3% in 2010 to 49.1% in 2021 caused a slight increase of 0.1% in under-5 mortality. A decrease in the proportion of children born with a birth order of 7 and above was observed between 2010 and 2021, likely attributable to a decline in fertility rates. The results indicate that 2.7% of the decline in fertility can be attributed to this decrease in the number (proportion) of children born with high birth orders. Concurrently, the subsequent increase in the proportion of firstborn children accounts for 0.9% of the decline. Lastly, a change in the proportion of children of birth order ranks 4 to 6 is responsible for an increase of 0.1%.

#### 3.3.2 Performance effect

Exploring the effects of coefficients in the evolution of mortality highlights the occupational status of the mother, the region of residence, and the birth order of the child as contributors to the evolution of under-five mortality. The occupational status of the mother is the only contributor to the decrease, with a contribution of around 13.2%. Changes in behavior were thus observed among unemployed women, leading to a more significant decrease in the mortality of their children compared to employed women. In addition, the variables of region of residence and birth order have a negative contribution to the decrease in mortality in terms of the effects of the coefficients. Indeed, while the other regions have experienced decreases in mortality similar to that of the Centre region (reference modality), those of the Cascades and Centre-Est have experienced less significant decreases, thus contributing negatively to the overall decline in mortality. Their contributions are, respectively, −2.5% and −8.0%. Similarly, children of rank 7 and above have a negative contribution of −5.1%. Mortality has seen a slight decrease in these children compared to the others, which, all things being equal, has contributed negatively to the overall decline (Table 4).

**Table 4:** Detailed results of the multivariate decomposition of the decline in under-five mortality.

		Differences due to characteristics			Differences due to coefficients		
Variables	Modality	Coef.	*p* > z	Pct.	Coef.	*p* > z	Pct.
Place of residence	Urban	Ref.	Ref.	Ref.	Ref.	Ref.	Ref.
	Rural	0.00200	0.003	4.50	0.00933	0.231	20.98
Region of residence	Boucle du Mouhoun	0.00000	0.988	0.01	−0.00053	0.714	−1.19
	Cascades	−0.00010	0.381	−0.22	−0.00109	0.054	−2.45
	Centre	Ref.	Ref.	Ref.	Ref.	Ref.	Ref.
	Centre−Est	0.00099	0.031	2.22	−0.00357	0.028	−8.04
	Centre−Nord	0.00022	0.326	0.50	−0.00037	0.794	−0.83
	Centre−Ouest	−0.00008	0.757	−0.18	0.00100	0.518	2.25
	Centre−Sud	−0.00001	0.914	−0.01	−0.00037	0.588	−0.83
	Est	0.00010	0.811	0.23	0.00039	0.810	0.87
	Hauts−Bassins	−0.00009	0.536	−0.21	−0.00143	0.282	−3.22
	Nord	0.00012	0.488	0.27	0.00102	0.291	2.29
	Plateau Central	−0.00007	0.745	−0.17	−0.00010	0.917	−0.23
	Sahel	0.00172	0.040	3.86	−0.00069	0.202	−1.56
	Sud−Ouest	0.00027	0.025	0.61	0.00013	0.873	0.30
Standard of living	Very Poor	0.00003	0.317	0.08	−0.00067	0.697	−1.52
	Poor	0.00005	0.438	0.11	0.00114	0.478	2.57
	Average	Ref.	Ref.	Ref.	Ref.	Ref.	Ref.
	Rich	−0.00004	0.502	−0.08	0.00179	0.289	4.01
	Very rich	0.00021	0.331	0.48	0.00042	0.841	0.95
Children of the household who slept under mosquito nets	None	−0.00078	0.328	−1.75	−0.00010	0.942	−0.22
	All	Ref.	Ref.	Ref.	Ref.	Ref.	Ref.
	Some	0.00098	0.006	2.20	−0.00074	0.605	−1.66
Mother’s education level	None	0.00235	0.028	5.28	0.00777	0.213	17.48
	Primary	0.00000	.	0.00	0.00000	.	0.00
	Secondary +	0.00135	0.385	3.03	−0.00084	0.707	−1.90
Mother’s religion	Muslim	−0.00021	0.075	−0.47	−0.00042	0.927	−0.94
	Christian	Ref.	Ref.	Ref.	Ref.	Ref.	Ref.
	Others	0.00068	0.102	1.54	−0.00009	0.862	−0.21
Mother’s ethnicity	Mossi	Ref.	Ref.	Ref.	Ref.	Ref.	Ref.
	Bobo/Dioula	0.00030	0.142	0.67	0.00074	0.205	1.67
	Fulfulde/peulh/Touareg/Bella	−0.00009	0.798	−0.19	0.00024	0.791	0.53
	Gourmantché	0.00078	0.007	1.75	0.00025	0.834	0.55
	Gourounsi	−0.00001	0.966	−0.02	−0.00021	0.808	−0.47
	Lobi/Dagara	0.00005	0.855	0.10	−0.00036	0.549	−0.81
	Senoufo	0.00039	0.191	0.88	0.00007	0.863	0.15
	Bissa	−0.00015	0.523	−0.33	0.00050	0.544	1.12
	Autres	−0.00027	0.602	−0.61	−0.00103	0.388	−2.31
Occupational status of mother	Inactive	−0.00239	0.008	−5.37	0.00586	0.011	13.18
	Active	Ref.	Ref.	Ref.	Ref.	Ref.	Ref.
Age group of mother at delivery	<20 years	0.00009	0.000	0.21	0.00043	0.752	0.96
	20–29 years	Ref.	Ref.	Ref.	Ref.	Ref.	Ref.
	30–39 years	0.00041	0.016	0.93	−0.00034	0.887	−0.77
	40–49 years	0.00001	0.812	0.02	−0.00030	0.639	−0.67
Sex of child	Male	Ref.	Ref.	Ref.	Ref.	Ref.	Ref.
	Female	−0.00002	0.058	−0.05	0.00288	0.254	6.48
Birth order of child	1	0.00039	0.051	0.89	0.00083	0.668	1.87
	2–3	Ref.	Ref.	Ref.	Ref.	Ref.	Ref.
	4–6	−0.00002	0.029	−0.05	−0.00132	0.593	−2.97
	7 +	0.00122	0.023	2.74	−0.00226	0.016	−5.09
	_cons				0.01611	0.327	36.23

Ultimately, the analysis shows that the decline in mortality is mainly attributable to a performance effect among unemployed women (13.1%), the composition effect associated with the decline in the proportion of uneducated mothers (5.3%), the decline in the number of households where no child sleeps under a mosquito net (2.2%) and urbanization (4.5%).

## 4 Discussion

The analysis of the results highlights the substantial contributory role of unobserved variables in this study. These unobserved variables, which are associated with policy performance and behavioral changes, had a significant effect on the reduction of under-five mortality rates between 2010 and 2021, accounting for nearly 77% of the observed decline. When considering the individual effects, it is evident that changes in the behavior of unemployed women contributed 13.1% to the decline, while enhancements in women’s education accounted for 5.3%. Additionally, government initiatives aimed at distributing mosquito nets to the majority of households resulted in increased utilization of these mosquito nets among children, contributing 2.2% to the decline. Urbanization also played a role, responsible for 4.5% of the decrease. Collectively, these factors reflect significant positive impacts on both economic and health development, ultimately leading to a decline in mortality rates among children under five.

Indeed, the mortality rate among children of unemployed mothers, which in 2010 was significantly higher, has experienced a marked decline, reaching levels comparable to those of children of working mothers in 2021. In addition, the increased utilization of mosquito nets among children has substantially decreased under-five mortality rates by reducing the prevalence of malaria, which continues to be the leading cause of death in the southern country. As noted in the contextual analysis, the prevalence of malaria in Burkina Faso saw a significant reduction between 2010 and 2021. Furthermore, the decline in mortality rates has also been influenced by urbanization. The migration of populations from rural areas to urban centers has facilitated the adoption of new lifestyles and improved access to health infrastructure, thereby contributing to a reduction in mortality risk for their offspring. However, it is important to note that overall, mortality among children under five is declining, even in non-urbanized areas. This is what Lankoandé and co-authors highlighted by analyzing inequalities and trends in under-five mortality in formal and informal settlements in Ouagadougou. They attribute the decline in inequalities in this mortality to the decrease in deaths from malaria and other causes, including sepsis, HIV/AIDS, measles, meningitis, and encephalitis (19).

For unobserved variables, it is important to recall the various health development and communication policies (with a view to driving social and behavioral changes) implemented by the country in recent years. For example, the health development process would have considered the integration of the empowerment of beneficiary communities in the management of their health problems (36). To this end, an important place was given to communication aimed at instilling a change in the health behaviors of populations at the local level. To do this, the promotional approaches used by Community-Based Health Workers (CBHAs) include awareness-raising through health education, care for people living with HIV (PLHIV), hygiene promotion, home visits, and the organization of populations to promote health (36). In addition to the action of Community-Based Health Workers, we can also mention the role of NGO actors in communication for behavior change in the various regions of the country. For example, some evaluated work shows how awareness campaigns can reduce child mortality (37). These actions could have had some success on the health of poor populations since the trend analysis of many health behavior indicators (vaccination completeness, prenatal and postnatal monitoring, use of health services) reveals improvements in uneducated populations and in poor households over the last few decades (see context analysis). Additionally, the implementation of major policies such as the free healthcare policy from 2016 would have had a probable effect on reducing mortality in this segment of the population by encouraging attendance at health centers, given the lifting of the financial barrier, something that has improved the health status of children.

Our results reinforce those of Demombynes et Trommlerova (38), who showed, also using an Oaxaca-Blinder decomposition, that the reinforcement of the use of insecticide-treated mosquito nets was a factor, if not the main factor, in the decline in infant mortality between 2003 and 2008 in Kenya. Also, these results align with the conclusions reached by Bassinga and Soura (26) through an analysis of the sources of change in under-five mortality between 1996 and 2006 in Burkina Faso. They also corroborate the findings of Adekunle (39), who mainly attributed the decline in under-five mortality at the regional level in Nigeria to changes in reproductive behaviors, particularly the lengthening of the inter-birth interval and the increase in the age at childbearing. As for the downward effects associated with changes in the composition of the population, they serve as a reminder of the importance of relying on levers such as population education and urban development (urbanization) to intuitively drive best practices in the fight against child mortality. The latter will undoubtedly involve the best hygiene practices, which are important for combating child mortality (17). Furthermore, investment efforts in health would be essential, as evidenced by Ayipe and Tanko (17), whose study revealed that for each additional percentage increase in national health expenditure, it is likely that there will be a decrease of about 5.3 units in the under-five mortality rate.

The results of this study provide significant lessons for improving child health in the country. A structured approach is needed to ensure sustainable progress. Strengthening public health policies must be a priority. The implementation of the free healthcare policy for children under five and pregnant women, introduced in 2016, has played a central role in reducing child mortality. To maintain this momentum, it is essential to sustain this initiative and expand its scope to eliminate financial barriers to access to care. Continuous improvement of obstetric and neonatal care, including antenatal and postnatal care, remains essential to prevent preventable deaths among newborns and young children.

Behavior change communication is another crucial lever. Awareness campaigns should be intensified, especially for unemployed women in rural areas. These campaigns have already proven effective in encouraging the adoption of practices beneficial to child health. The promotion of the systematic use of insecticide-treated mosquito nets must also be strengthened. This measure has contributed to the reduction of malaria-related deaths, a major cause of child mortality in Burkina Faso.

Maternal education and women’s empowerment are determining factors. The reduction in the number of uneducated mothers has led to a significant decrease in child mortality. Investing in the education of girls and women remains a priority to ensure a lasting impact on children’s health. Programs to support women’s economic empowerment can improve the living conditions of families and, consequently, the health of children.

The process of urbanization and the improvement of health infrastructure also play a significant role. Migration to urban centers has led to a decrease in child mortality thanks to better access to health services. However, it is imperative to ensure that health infrastructure in rural areas also benefits from this development to reduce disparities. Specific interventions need to be implemented in regions such as the Sahel and the South-West, which have high mortality rates.

To maximize the impact of these interventions, a multisectoral approach is essential. The fight against child mortality is not only the responsibility of the health sector but also relies on advances in education, economic development, and sanitation. Intersectoral coordination helps to harmonize efforts and ensure a comprehensive and coherent response. In addition, the engagement of local communities and non-governmental actors in the planning and implementation of interventions ensures solutions adapted to the realities on the ground.

Finally, strengthening data collection and analysis is crucial to effectively guiding public policies. Continuous monitoring, through demographic and health surveys (DHS), makes it possible to evaluate interventions and adjust strategies according to identified needs. A thorough understanding of unobserved factors, such as behavioral changes and the effectiveness of health systems, is necessary to refine these policies.

### 4.1 Limitations of the study

This study, although offering very interesting results, has some limitations that must be highlighted. Despite their advantages, the DHS data used in this analysis have some limitations inherent in any retrospective, cross-sectional, single-pass survey, including selection bias and omissions. Indeed, only survivors aged 15–49 who had not migrated at the time of the survey were interviewed about the survival and health of their children. This implicitly assumes that the phenomenon studied (infant mortality in the case of this study) and disruptive events are independent. However, if the number of children whose mothers are not alive or have emigrated is large, or if they have a different mortality rate than children whose mothers are interviewed, this would result in a significant bias in the estimation of mortality.

In addition, by using the respondents’ residence at the time of the survey to calculate the indicators, it is implicitly assumed that births and deaths occur in the region and area of residence of the respondents at the time of the survey. However, these events may have occurred in the original residence, especially for recent migrations. Thus, if rural-to-urban migrations are significant and if infant mortality is higher in rural areas, and a significant proportion of these deaths occur in rural areas before the mother’s migration to urban areas, this can create a significant bias in the difference in mortality between rural and urban areas (40).

Also, the characteristics and living conditions of households and mothers may have changed over the past 5 years to the point that they are not the same between the occurrence of deaths or births and the time of the survey. An analysis of infant mortality based on the characteristics and living conditions of households and mothers at the time of the survey, therefore, assumes that these have not changed significantly between the death of the child and the time of the survey.

DHSs draw on memory to restore relatively distant events. This can then result in risks of omission, imprecision, and concordance in the dating of events, leading to an underestimation of infant mortality, for example (41). However, according to Sullivan, Bicego, and Rutstein (42), the methodological limitations inherent in birth history and the risks of errors or imprecision in collection generally result in only a very small margin of error in the measurements of recent events. Additionally, questions on health status and health services, such as vaccinations, were only asked for surviving children (not for all children) born in the 5 years prior to the survey and are therefore not applicable in the multivariate model. Symptoms of acute respiratory infection (ARI), fever, and diarrhea, as well as their treatment, were restricted to the two-week period preceding the interview among surviving children born in the last 5 years and are therefore not applicable either. Similarly, some essential variables, such as spouse’s education and spouse’s occupation, were not included due to a high level of non-response.

With regard to the Oaxaca-Blinder decomposition, while robust, it assumes linearity and additive effects, which may oversimplify complex interactions between socioeconomic, behavioral, and policy factors. Unobserved variables, such as localized healthcare quality, cultural shifts in care-seeking behaviors, or community-level health initiatives, were not captured in the model, potentially inflating the attributed contribution of “performance effects.” For instance, the free healthcare policy implemented in 2016 likely influenced healthcare access, but granular data on policy rollout or regional disparities in implementation were unavailable, limiting our ability to disentangle its specific impact.

These limitations underscore the need for longitudinal data and mixed-method approaches to better capture dynamic interactions between structural, behavioral, and policy drivers of child survival in resource-limited settings.

## 5 Conclusion

This analysis aimed to identify the sources of change in under-five mortality between 2010 and 2021 based on data from demographic and health surveys. Indeed, infant and child mortality has fallen considerably over this period. In 2010, out of a thousand live births, 128.4 died before celebrating their fifth birthday. This risk of death is 47.9 per thousand live births in 2021, a decrease of 80.5 points. Our findings indicate that 77% of the reduction can be attributed to performance effects, encompassing behavioral changes and the efficacy of health policies, while 23% is associated with shifts in population composition. The analyses show that the effects of performance and behavioral changes, certainly driven by political efforts in terms of health provision and improvement of health behaviors, have had a more significant impact on the observed decline process. Also, in terms of performance and behavioral changes, it is necessary to note the action of unemployed women (13.1%) who have managed to significantly reduce their mortality level. In terms of structural or compositional effects, the decline in the number of uneducated women (with an effect of 5.3%), the decline in households where no child sleeps under a mosquito net (2.2%) and urbanization (4.5%) were determining factors in the observed decline.

However, persistent disparities in high-risk regions like the Sahel and Southwest demand targeted interventions to improve rural healthcare access, maternal education, and economic opportunities. Urbanization, while beneficial, must be complemented by equitable infrastructure development to bridge urban–rural gaps.

These results highlight the need to address the mortality bias with holistic interventions that integrate economic and health development efforts, as well as education and awareness through behavior change communication. Strengthening community health worker programs, expanding mosquito net distribution, and prioritizing girls’ education will amplify gains. Policymakers must also leverage data-driven strategies to address region-specific challenges and ensure no child is left behind. Sustained investment in these areas, coupled with robust monitoring, will solidify Burkina Faso’s trajectory toward eliminating preventable child deaths and achieving health equity.

## Data Availability

Publicly available datasets were analyzed in this study. This data can be found here: https://dhsprogram.com/Countries/Country-Main.cfm?ctry_id=50&c=Burkina%20Faso&Country=Burkina%20Faso&cn=&r=1.

## Footnotes

^1^One of the limitations of the decomposition method used here is that it does not provide an R^2^ type indicator to measure the share of variance explained by the decomposition.
